# Effector and Central Memory Poly-Functional CD4^+^ and CD8^+^ T Cells are Boosted upon ZOSTAVAX^®^ Vaccination

**DOI:** 10.3389/fimmu.2015.00553

**Published:** 2015-10-29

**Authors:** Janet J. Sei, Kara S. Cox, Sheri A. Dubey, Joseph M. Antonello, David L. Krah, Danilo R. Casimiro, Kalpit A. Vora

**Affiliations:** ^1^Merck Research Laboratories, Department Vaccine Analytical Development, Merck & Co., Inc., Kenilworth, NJ, USA; ^2^Merck Research Laboratories, Department of Infectious Diseases and Vaccines, Merck & Co., Inc., Kenilworth, NJ, USA

**Keywords:** ZOSTAVAX, VZV antigens, poly-functional T cells, flow cytometry, memory response

## Abstract

ZOSTAVAX^®^ is a live attenuated varicella-zoster virus (VZV) vaccine that is licensed for the protection of individuals ≥50 years against shingles and its most common complication, postherpetic neuralgia. While IFNγ responses increase upon vaccination, the quality of the T cell response has not been elucidated. By using polychromatic flow cytometry, we characterized the breadth, magnitude, and quality of *ex vivo* CD4^+^ and CD8^+^ T cell responses induced 3–4 weeks after ZOSTAVAX vaccination of healthy adults. We show, for the first time that the highest frequencies of VZV-specific CD4^+^ T cells were poly-functional CD154^+^IFNγ^+^IL-2^+^TNFα^+^ cells, which were boosted upon vaccination. The CD4^+^ T cells were broadly reactive to several VZV proteins, with immediate early (IE) 63 ranking the highest among them in the fold rise of poly-functional cells, followed by IE62, gB, open reading frame (ORF) 9, and gE. We identified a novel poly-functional ORF9-specific CD8^+^ T cell population in 62% of the subjects, and these were boosted upon vaccination. Poly-functional CD4^+^ and CD8^+^ T cells produced significantly higher levels of IFNγ, IL-2, and TNFα compared to mono-functional cells. After vaccination, a boost in the expression of IFNγ by poly-functional IE63- and ORF9-specific CD4^+^ T cells and IFNγ, IL-2, and TNFα by ORF9-specific poly-functional CD8^+^ T cells was observed. Responding poly-functional T cells exhibited both effector (CCR7^−^CD45RA^−^CD45RO^+^), and central (CCR7^+^CD45RA^−^CD45RO^+^) memory phenotypes, which expressed comparable levels of cytokines. Altogether, our studies demonstrate that a boost in memory poly-functional CD4^+^ T cells and ORF9-specific CD8^+^ T cells may contribute toward ZOSTAVAX efficacy.

## Introduction

Herpes zoster (HZ) or shingles is a debilitating disease characterized by a vesicular rash, with the most common complication being postherpetic neuralgia (PHN). PHN is a constant and severe pain that develops after clearance of the cutaneous outbreak and can last for several years, thereby contributing to the high morbidity of affected individuals (1). HZ is caused by reactivation of latent varicella-zoster virus (VZV) from the sensory ganglia (2). Immune responses generated during primary VZV infection (chickenpox) have been shown to prevent the reactivation of latent VZV (3, 4). However, the incidence of HZ is strongly associated with advancing age (5, 6). Several investigations have shown that T cell-mediated immune responses decline with increasing age and during immunosuppression, resulting in reactivation of VZV (6–11). However, the levels of anti-VZV antibodies remain relatively stable with increasing age (6, 12, 13), demonstrating that the humoral immune response may not be sufficient for the prevention of HZ. Several studies have reported the induction of VZV-specific CD4^+^ and CD8^+^ T cells (14–20), with CD4^+^ T cells dominating the memory response (18, 19, 21–23).

The HZ vaccine (ZOSTAVAX^®^) is a live attenuated Oka/Merck VZV vaccine indicated for the prevention of HZ and its complications in individuals 50 years and older and is approved in many countries around the world. ZOSTAVAX reduced the incidence of HZ by 69.8% among people aged 50–59 years (24) and diminished PHN by 66.5% in people ≥60 years old (25). ZOSTAVAX vaccination led to a fold rise in VZV antibody levels (13, 26, 27) and a fold increase in VZV-specific effector memory and IFNγ-producing cells (9, 13, 26).

The VZV antigens that are known to induce T cell-mediated immune responses include structural glycoproteins gB, gC, gE, gH, gI, immediate early (IE) 4, IE62, and IE63 proteins and open reading frame (ORF) 10 tegument protein (14–20). However, the aforementioned studies focused on individual set(s) of viral proteins (single or limited numbers of proteins in each study). Hence, a comprehensive analysis of the immunodominant T cell antigens involved in protection against HZ, and following ZOSTAVAX vaccination warrants further investigation. To this end, Laing et al. showed that the diversity of the VZV-specific CD4^+^ T cell repertoire increases after ZOSTAVAX vaccination (28). A month after vaccination, the authors observed an increase in CD4^+^ T cells that were reactive to glycoproteins (gI, gE, and gH), IE proteins (IE63 and IE62), tegument proteins (ORF4, ORF9, ORF10, and ORF12), capsid proteins (ORF40 and ORF41), and enzymes (ORF18, ORF36, ORF37, and ORF59) (28). However, the continuous antigenic stimulation required to establish VZV-reactive T cell lines could lead to a sampling bias as antigen-specific T cells with the highest frequencies may be over-represented. Consequently, we sought to investigate the *ex vivo* (with minimal *in vitro* manipulation) VZV-specific T cell responses induced upon ZOSTAVAX vaccination. Moreover, the quality of the T cell response as represented by poly-functionality, cytokine expression, and memory phenotype has been shown to correlate with vaccine efficacy for other pathogens: hence, we investigated whether these responses were induced upon ZOSTAVAX vaccination.

Our results independently confirm the data from Laing et al. (28) that ZOSTAVAX vaccination leads to broad T cell responses. We further demonstrate, for the first time that the highest frequencies of memory VZV-specific CD4^+^ T cells in older subjects express four functions (CD154^+^IFNγ^+^IL-2^+^TNFα^+^). Upon vaccination, there was a fold increase in the frequency of antigen-specific poly-functional CD4^+^ T cells. Of the VZV antigens assessed, IE63 was dominant in stimulating the highest fold rise in the frequency of CD154^+^IFNγ^+^IL-2^+^TNFα^+^ CD4 T cells after vaccination. In 62% of the subjects, we identified a novel poly-functional CD8^+^ T cell population that was reactive to ORF9 protein and was boosted upon vaccination. On a per-cell basis, poly-functional CD4^+^ and CD8^+^ T cells expressed ~10× higher levels of IFNγ, IL-2, and TNFα than mono-functional T cells. After vaccination, we observed statistically significant increases in the expression of IFNγ by IE63- and ORF9-specific poly-functional CD4^+^ T cells, and IFNγ, IL-2, and TNFα expressions by poly-functional ORF9-specific CD8^+^ T cells. Lastly, phenotypic characterization of antigen-specific memory T cells demonstrated that they belonged to both central and effector populations. Based on our findings, we hypothesize that IE63 and ORF9 proteins are key VZV antigens required for protective responses following ZOSTAVAX vaccination.

## Materials and Methods

### Peptide Design

Sequences for all antigens except IE62 are from VZV parental Oka strain accession number AB097933.1 (29). The sequence for IE62 is from the VZV vaccine Oka strain accession number AB097932.1 (29). Synthetic peptides (JPT, Germany) were synthesized as 15-mers overlapping by 11 amino acids, with >70% purity. The majority of impurities in synthetic peptide preparations result from the formation of peptides with shorter sequences compared to the parental target sequence. These truncated sequences do not cause non-specific stimulation because they are of the same amino acid sequence. Peptide pools were limited to a maximum of 120 sequences per pool. For larger proteins, we generated two or three peptide pools: gB-1_1–439_, gB-2_429–743_, gE-1_1–319_, gE-2_309–623_, IE62-1_1–443_, IE62-2_433–875_, IE62-3_865–1,310_, ORF19-1_1–96_, ORF19-2_97–191_, ORF29-1_1–407_, ORF29-2_397–803_, and ORF29-3_793–1,199_. Lyophilized peptides were solubilized in dimethyl sulfoxide (DMSO, Sigma, St. Louis, MO, USA) at 20–50 mg/ml and pooled within each protein so that the concentration of each peptide in the pool was 400 μg/ml. Peptide pools were stored at −70°C in small aliquots to limit freezing and thawing cycles. Consistent with clinically validated assays used to quantify antigen-specific responses after vaccination, DMSO was used as a negative control (30–34). VZV lysate was generated by propagating VZV Oka/Merck vaccine strain in human embryonic lung fibroblasts MRC5 cell line. Infected cells were harvested upon observation of cytopathic effect, lysed by sonication, and UV treated to inactivate the virus. Prior to UV irradiation, the titer of the VZV stock was determined by plaque reduction assay. VZV lysate was aliquoted and cryopreserved at −70°C. For VZV lysate-specific responses, MRC5 lysate was used as a negative control.

### Human Subjects

The Merck Institutional Review Board approved the human studies, and all subjects provided written informed consent. Merck employees who requested ZOSTAVAX vaccination were approached about volunteering for this study. Twenty-one subjects (12 male and nine female) between 55 and 65 years with a history of chickenpox were vaccinated with ZOSTAVAX (Merck Research Laboratories, Merck & Co., Inc., Kenilworth, NJ, USA). Blood was drawn from the subjects prior to vaccination and 3–4 weeks after vaccination. The selection of this time point was based on several of the clinical trial protocols that led to the licensure of ZOSTAVAX (13, 26). This time point was also independently shown to be a peak response time for T cell responses by Laing et al. (28).

### Whole Blood Stimulation and Cytokine Release Assay

Whole blood was collected in sodium heparin vacutainer tubes and used for peptide, MRC5 lysate, and VZV lysate stimulation within 2 h of the blood draw. For each stimulation condition, 1 ml of blood was incubated with a final concentration of 2.5 μg/ml of each peptide within a pool. A matching volume of DMSO was used as a negative control sample for each subject. Blood was incubated at 37°C for 24 (±2) h. Blood tubes were then centrifuged at 900 × *g* for 10 min to separate the plasma. Plasma was harvested, aliquoted, and stored at −70°C until the time of the assay. The plasma was tested for cytokines with electrochemiluminescent cytokine assay kits from Meso Scale Discovery (Rockville, MD, USA) [Human Pro-Inflammatory-4 I Plex ultrasensitive kit (catalog number K15009C-2) and Human TH1/TH2 7-Plex ultrasensitive kit (catalog number K15011C-2)] according to the manufacturer’s instructions. Plates were read on an MSD sector Imager 2400 and the picogram per milliliter of each cytokine was calculated with MSD software four-paramater fit curve. Non-stimulated (background) cytokine levels were subtracted from all stimulated sample results.

### PBMC Isolation

PBMC were isolated and cryopreserved as previously described (35). Frozen PBMC were thawed in complete RPMI-1640 (Invitrogen), washed and rested overnight in a 37°C/5% CO_2_ incubator. Complete RPMI-1640 contains 55 μM 2-mercaptoethanol (Invitrogen), 10 mM HEPES buffer (Invitrogen), 2 mM l-glutamine (Invitrogen), 1 mM MEM sodium pyruvate solution (Invitrogen), 1× penicillin–streptomycin solution (Invitrogen), and 10% heat-inactivated FBS.

### Antigen Stimulation and Intracellular Cytokine Staining Assays

The OMIP-014-validated assay (33) was adapted for our studies. Rested PBMC at 1 × 10^6^ PBMC/well were incubated with peptide pools at a final concentration of 2 μg/ml per peptide for 5.5 h in a 37°C/5% CO_2_ incubator, in the presence of 1.25 μg/ml anti-CD28 (BD Biosciences, San Jose, CA, USA) and anti-CD49d (BD Biosciences) costimulatory antibodies. Brefeldin A (Sigma) and monensin (Sigma) both at 5 μg/ml final concentrations were added after the initial 30 min of incubation. As positive controls, PBMC were stimulated with a predetermined titer of VZV Oka lysate and *staphylococcal enterotoxin* B (SEB: 3 μg/ml, Sigma). Negative controls cells were treated with DMSO and uninfected MRC5 cell lysate. Following incubation, 20 μl of 20 mM EDTA (Sigma) was added, and the 96-well plate was transferred to 4°C overnight. Overnight incubation with EDTA yielded similar staining patterns as when cells were stimulated and stained in 1 day.

The next day, cells were washed with 1× PBS and stained with LiveDead Violet kit (Invitrogen) for 20 min at room temperature. Cells were washed with FACS wash [1× PBS + 1% FBS + 0.01% sodium azide (Sigma)], permeabilized, and fixed in BD cytofix/cytoperm (BD Biosciences) for 20 min at 4°C. PBMC were stained with a cocktail of the following antihuman monoclonal conjugated antibodies for 30 min at room temperature: CD3-ECD (clone UCHT1, Beckman Coulter, Brea, CA, USA), CD8-PerCP-Cy5.5 (clone SK1, BD Biosciences), CD4-APC-Alexafluor 750 (clone 13B8.2, Beckman Coulter), IL-2-APC (clone 534.111, BD Biosciences), IFNγ-BV500 (clone B27, BD Biosciences), TNFα-PE-Cy7 (clone Mab11, BD Biosciences), Perforin-FITC (clone B-D48, Cell Sciences, Canton, MA, USA), CD154-PE-Cy5 (clone TRAP1, BD Biosciences), and IL-10-PE (clone JES3-19F1, BD Biosciences). After washing with FACS wash, the cells were resuspended in BD-stabilizing fix (BD Biosciences) until flow cytometric analysis.

For staining of memory markers and cytokines, the cells were incubated with the following antihuman antibodies for 45 min at 4°C, prior to permeabilization and fixation: CCR7-FITC (clone G043H7, Biolegend, San Diego, CA, USA), CD45RO-PE (clone UCHL1, eBiosciences, San Diego, CA), and CD45RA-Brilliant Violet 605 (clone HI100, Biolegend). The protocol described earlier was then followed with a cocktail of CD3-ECD, CD8-PerCP-Cy5.5, CD4-APC-Alexafluor 750, IL-2-APC, IFNγ-BV500, TNFα-PE-Cy7, and CD154-PE-Cy5.

### Flow Cytometric Analysis

Samples were analyzed on an LSR II (BD Biosciences), after instrument standardization and characterization by using the BD Cytometer Setup and Tracking (CS&T) software and BD™ CS&T beads (BD Biosciences). Compensation was performed using the BD FACS Diva software (BD Biosciences) based on OneComp beads (eBiosciences) stained for each individual antibody, and ArC™ Amine Reactive Compensation Bead Kit (Invitrogen) for viability dye staining. Between 400,000 and 800,000 events were collected for each sample. Analysis was performed using FlowJo software v9.8.2 (TreeStar Inc., Ashland, OR, USA). DMSO background levels were subtracted from peptide-stimulated samples, and MRC5 lysate was subtracted from VZV lysate.

Presentation analysis of antigen-specific heterogeneous T cell distribution was performed using the Simplified Presentation of Incredibly Complex Evaluations’ program version 5.35, downloaded from <http://exon.niaid.nih.gov/spice> (36). The number of antigen-specific T cells was calculated by multiplying the frequency of cells producing cytokines by 10^6^ and dividing this value by the frequency of cells in the lymphocyte gate. In cases where the background had higher events than the positive, resulting in negative values, these were corrected to 1 to allow calculation of fold rise.

### Statistical Analysis

Differences in cytokine levels prior to and following ZOSTAVAX administration in the 21 subjects were assessed for alternative peptide pools using an Exact Wilcoxon’s Signed Rank Test. The analyses were performed using StatXact (Cambridge, MA, USA) version 4.0.1 for Windows NT. The Exact Wilcoxon’s Signed Rank Test is appropriate for identifying a statistically significant increase in T cell responses after vaccination, relative to responses before vaccination in the same subject. The purpose of the analysis was to provide a metric for the rank ordering of the boost in response across the set of different antigens. The one-sided *P* values from the Exact Wilcoxon’s Signed Rank Test serve as that metric. These analyses are summarized in Tables 1 and 2. Cytokine expression levels in poly-functional and mono-functional cells prior to and following ZOSTAVAX vaccination were statistically compared by two-tailed paired Student’s *t*-test performed using Prism software (GraphPad, La Jolla, CA, USA). Significant differences were identified by *P* values: not significant *P* ≥ 0.05, *P ≤ 0.05, **P ≤ 0.01, ***P ≤ 0.001, and ****P ≤ 0.0001.

**Table 1:** **Hierarchy of VZV antigens stimulating IFNγ, IL-2, and TNFα**.

IFNγ			IL-2			TNFα		
Antigen rank	Fold rise	One-sided *P*-value	Antigen rank	Fold rise	One-sided *P*-value	Antigen rank	Fold rise	One-sided *P*-value
IE62-1	2.94	0.0000	IE62-2	3.38	0.0000	gB-1	2.29	0.0031
IE62-2	4.41	0.0000	IE62-1	2.08	0.0002	ORF9	1.61	0.0273
gB-2	2.30	0.0002	IE62-3	2.40	0.0004	IE62-2	1.79	0.0323
ORF9	4.02	0.0002	IE63	2.35	0.0005	gB-2	1.16	0.0448
gE-2	3.31	0.0007	ORF9	3.23	0.0019	ORF4	0.82	0.0516
IE62-3	2.79	0.0008	gB-2	2.27	0.0021	IE63	1.89	0.1128
IE63	4.31	0.0008	gE-2	2.94	0.0024	IE62-3	1.50	0.1214
gB-1	2.95	0.0012	gE-1	2.69	0.0031	ORF19-1	0.89	0.1623
ORF19-1	1.36	0.0017	ORF4	2.24	0.0097	gE-1	1.24	0.1666
gE-1	2.99	0.0021	gB-1	1.45	0.0210	IE62-1	1.49	0.1688
ORF10	1.75	0.0032	ORF19-1	1.38	0.0301	ORF10	0.83	0.2060
ORF29-2	1.78	0.0053	ORF29-1	1.71	0.0399	IE61	0.92	0.2584
ORF4	2.91	0.0071	ORF19-2	1.24	0.0895	gE-2	1.33	0.4204
ORF29-3	3.32	0.0107	ORF29-3	1.11	0.2375	ORF19-2	1.03	0.4324
ORF29-1	1.41	0.0223	ORF10	1.03	0.2899	ORF29-1	1.06	0.4459
ORF19-2	1.69	0.0487	ORF29-2	1.00	0.3047	ORF29-2	0.97	0.4459
IE61	1.17	0.0567	IE61	1.09	0.3166	ORF29-3	0.90	0.4459

*P values were calculated using an Exact Wilcoxon’s Signed Rank Test*.

**Table 2:** **Ranking of VZV antigens stimulating poly-functional CD4^+^ T cells**.

4+ functions (CD154^+^IFNγ^+^IL-2^+^TNFα^+^)			3+ functions (CD154^−^IFNγ^+^IL-2^+^TNFα^+^)		
VZV antigen	Geometric mean fold rise	One-sided *P*-value	VZV antigen	Geometric mean fold rise	One-sided *P*-value
IE63	3.66	<0.0001	IE62-2	2.61	<0.0001
IE62-3	2.25	0.0001	IE62-3	3.50	0.0001
gB-1	2.73	0.0005	ORF9	3.03	0.0001
VZV lysate	2.43	0.0005	gE-1	3.09	0.0002
ORF9	2.64	0.0011	gB-2	2.42	0.0009
gE-1	2.24	0.0012	IE62-1	1.75	0.0040
IE62-1	2.40	0.0020	IE63	2.28	0.0060
IE62-2	2.21	0.0036	gE-2	2.25	0.0101
gE-2	1.95	0.0064	gB-1	2.75	0.0107
gB-2	2.02	0.0134	VZV lysate	2.09	0.0198

*P values were calculated using an Exact Wilcoxon’s Signed Rank Test*.

## Results

### Selection of VZV Antigens

Naturally acquired CMV infection elicits broad and strong T cell responses with 151 out of 231 ORF being immunogenic against CD4 and/or CD8 T cells (37). Immunogenicity was largely influenced by the abundance of the ORF in the virion and modestly by the expression kinetics or function of the ORF (37). Similarly, in a herpes simplex virus (HSV)-1 study, majority of the T cell responses were weighted toward IE proteins and abundant virus polypeptides (38). VZV has a 125 kb genome with 71 ORFs (39). Therefore, to narrow our focus on antigens that may be critical for anti-VZV responses, the following selection criteria were considered: T cell reactivity of homologs expressed by related herpes viruses that lead to the activation of both CD4 and CD8 T cells, the temporal and kinetic expression of the proteins, and the abundance of the proteins on the virion. As such, 10 ORFs of the VZV genome that have previously been demonstrated to induce VZV-specific T cell-mediated responses (14, 18, 19, 22, 40–46) or stimulate T cell responses in related herpes viruses (38, 47–49), or that were abundant during primary VZV infection or latency (15, 50, 51), were selected for this study – gB, gE, IE61, IE62, IE63, ORF4, ORF9, ORF10, ORF19, and ORF29.

### A Boost in IFNγ, IL-2, and TNFα Observed Following ZOSTAVAX Vaccination, in Response to Broad VZV Antigens

**Figure 1:**
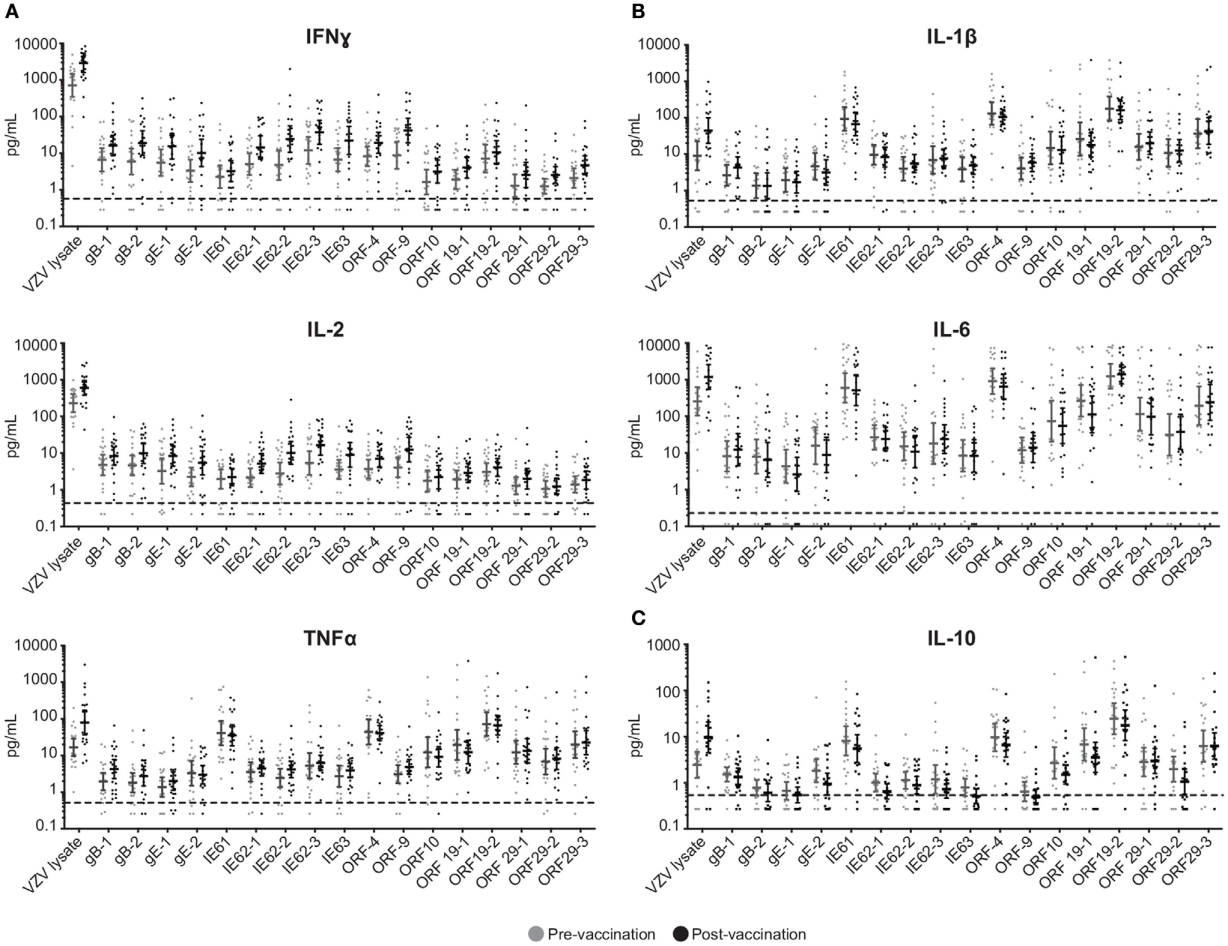
**Boost in IFNγ, IL-2, and TNFα following ZOSTAVAX vaccination**. Whole blood was drawn from 21 subjects prior to and following ZOSTAVAX (3–4 weeks) vaccination. Within 2 h of blood draw, the specimen was stimulated with VZV peptide pools, VZV lysate (positive control), MRC5, and DMSO (negative controls) for 24 h. Plasma was then harvested and tested for the presence of **(A)** effector cytokines – IFNγ, IL-2, and TNFα; **(B)** pro-inflammatory cytokines – IL-1β and IL-10; and **(C)** anti-inflammatory cytokine IL-10, by using the Meso Scale Discovery kits. Each dot represents one subject. The geometric mean ± 95% confidence interval is shown for all data sets. Horizontal broken line denotes the lowest limit of detection for all cytokines.

**Figure 2:**
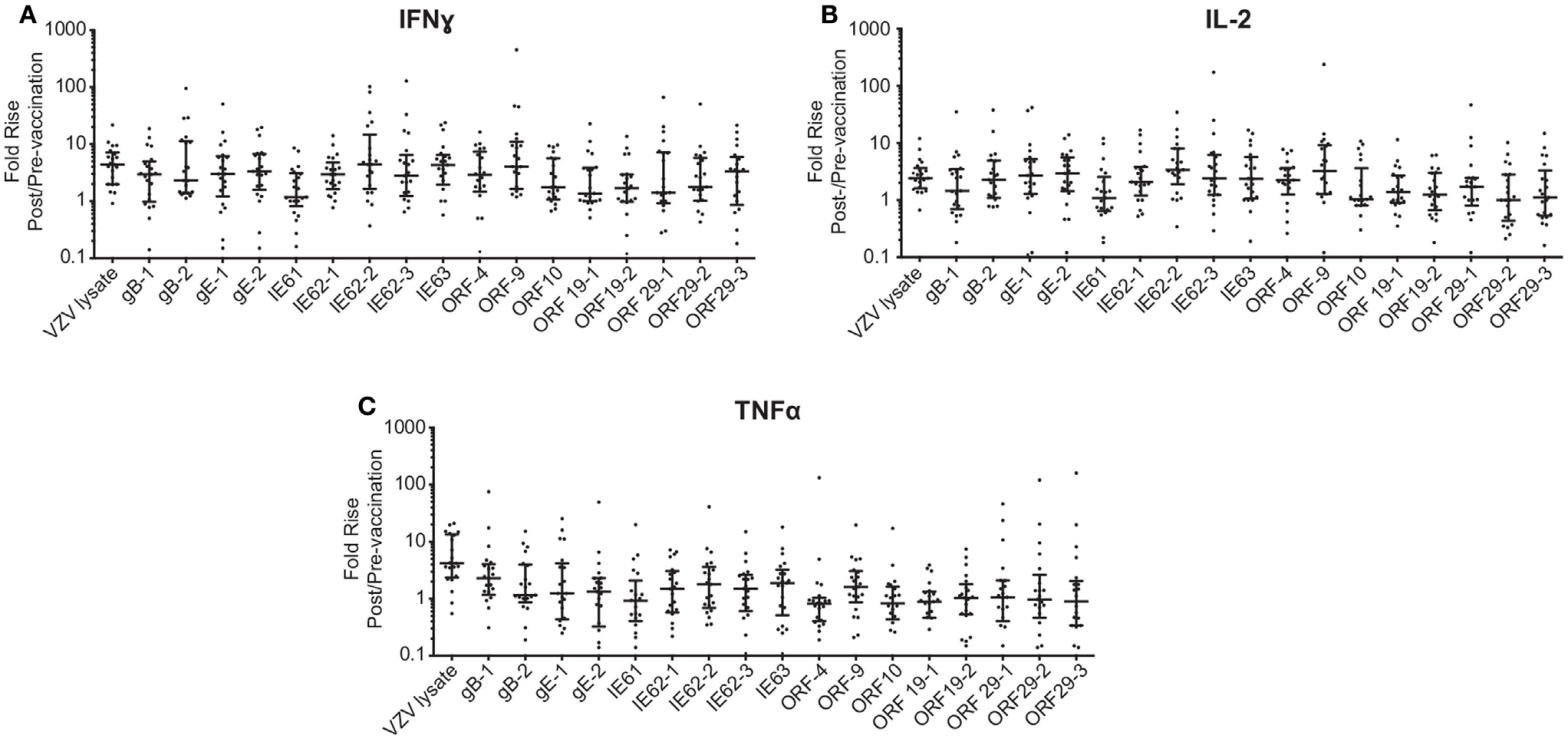
**Fold rise in **(A)** IFNγ, **(B)** IL-2, and **(C)** TNFα levels following ZOSTAVAX vaccination**. The fold rise (post/pre) was calculated for each vaccinated subject. Graph shows the median ± interquartile range for all 21 subjects.

To investigate the cell-mediated responses induced by ZOSTAVAX, peptide pools spanning the VZV proteins listed earlier were used to stimulate whole blood acquired from human volunteers prior to ZOSTAVAX administration and 3–4 weeks postvaccination. Baseline levels of cytokines were detected upon antigen stimulation of the prevaccination samples, demonstrating the presence of memory anti-VZV cells before vaccination (Figure 1). After ZOSTAVAX vaccination, we observed an increase in IFNγ, and IL-2 upon stimulation with all VZV antigens (Figure 1A). The increase in TNFα levels was not as robust as IFNγ and IL-2 and was only observed upon stimulation with gB, gE, IE62, IE63, ORF9, and ORF29 (Figure 1A). While baseline levels of pro-inflammatory responses (IL-1β and IL-6) were observed upon antigen stimulation prevaccination, vaccination with ZOSTAVAX did not boost their level of production (Figure 1B). Notably, a reduction in IL-10 levels was observed in peptide-stimulated whole blood following vaccination with ZOSTAVAX (Figure 1C). For all VZV antigens tested, the levels of IL-4 and IL-5 were below the limit of detection and did not increase after vaccination with ZOSTAVAX (data not shown). Fold increases in IFNγ, IL-2, or TNFα production upon antigen stimulation are shown in Figures 2A–C. Statistical analyses of VZV antigen pools stimulating fold increase in cytokine responses are summarized in Table 1. Overall, the peptide pools that stimulated robust levels of IFNγ, IL-2, and TNFα were gB-1, gB-2, gE-1, gE-2, IE62-1, IE62-2, IE62-3, IE63, and ORF9. Henceforth, these peptide pools were used as antigenic stimuli for further T cell investigations via flow cytometry.

### Fold Rise in Poly-Functional Antigen-Specific CD4^+^ T Cells Observed after Vaccination with ZOSTAVAX

**Figure 3:**
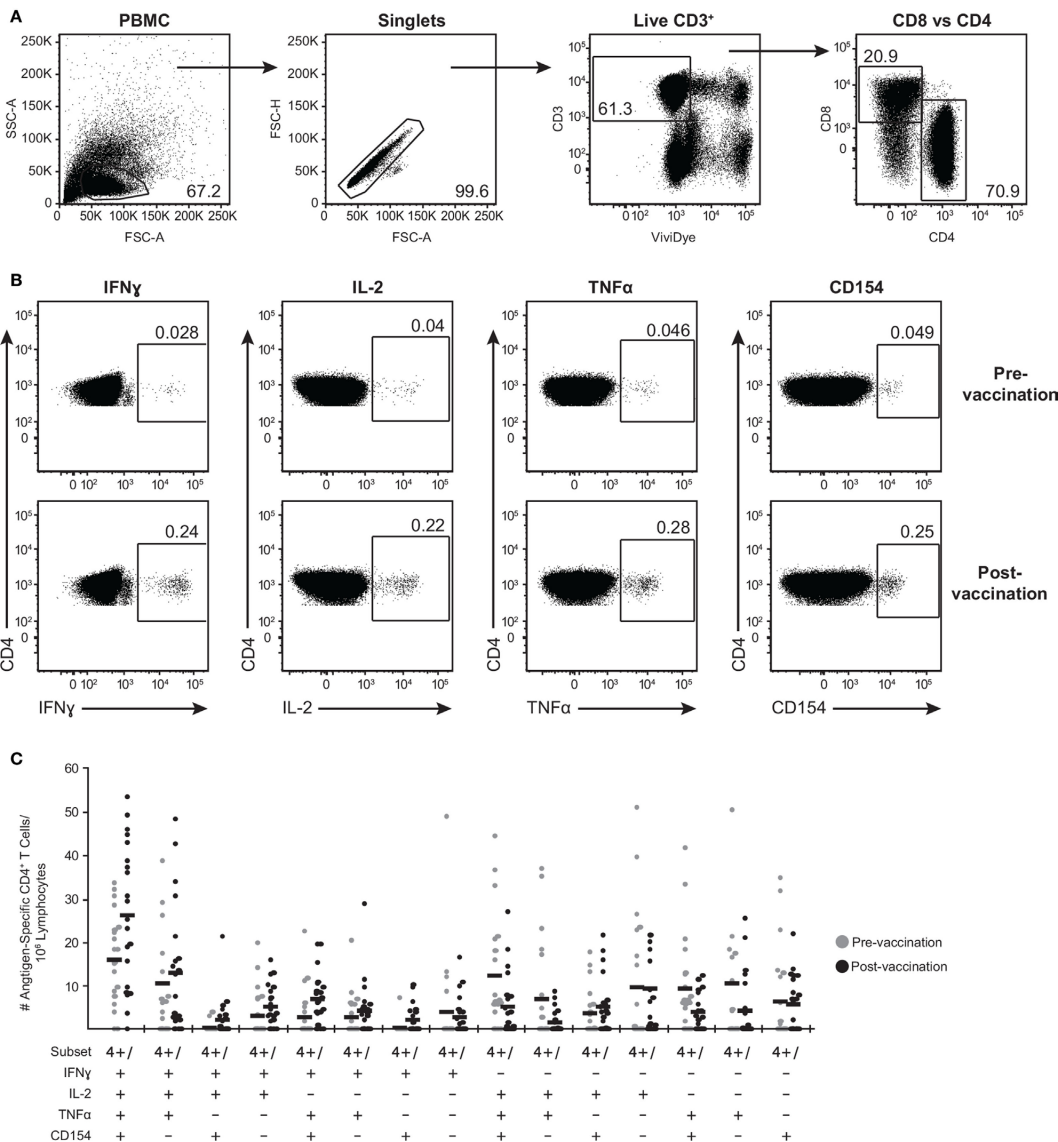
**Poly-functional CD4^+^ T cells are boosted following ZOSTAVAX vaccination**. PBMC were thawed and rested overnight before VZV antigen stimulation. One million PBMC/sample were stimulated with antigens for 5.5 h at 37°C/5% CO_2_, with brefeldin A and monensin added 30 min after the initial incubation. Cells were stained with Vividye, then fixed, permeabilized, and stained with antibodies to identify **(A)** CD3^+^ T cells, expressing either CD4 or CD8 that were producing **(B)** IFNγ, IL-2, TNFα, and CD154. Figure shows IE62-specific responses from one individual, and numbers on dot plots represent percentages of cells (DMSO subtracted) expressing each molecule. **(C)** Shows the heterogeneity of IE63-specific poly-functional CD4^+^ T cells for all 21 subjects as determined by Boolean-gating strategy. Each dot represents one subject, and the mean is shown for all phenotypes.

We further expanded the whole blood stimulation data by phenotyping the antigen-specific T cell-mediated responses in these subjects. PBMC were stimulated with the above-selected peptide pools, VZV lysate, MRC5 lysate, vehicle (DMSO), or SEB and analyzed by flow cytometry for viable CD3^+^ T cells expressing either CD4 or CD8 (Figure 3A) and intracellular IFNγ, IL-2, and TNFα (Figure 3B). To identify activated CD4^+^ T cells, we also assessed for *de novo* expression of CD154 (CD40L) (52) (Figure 3B). A representative gating strategy from one individual is shown in Figures 3A,B. In this subject, low frequency of memory CD4^+^ T cells eliciting each of the four functions upon stimulation with IE62 was detectable prior to vaccination (Figure 3A). Following vaccination, a boost in frequency of CD4^+^ T cells producing IFNγ^+^ increased by 8.6-fold, IL-2^+^ increased by 5.5-fold, TNFα^+^ increased by 6.1-fold, and CD154^+^ increased by 5.1-fold (Figure 3A).

**Figure 4:**
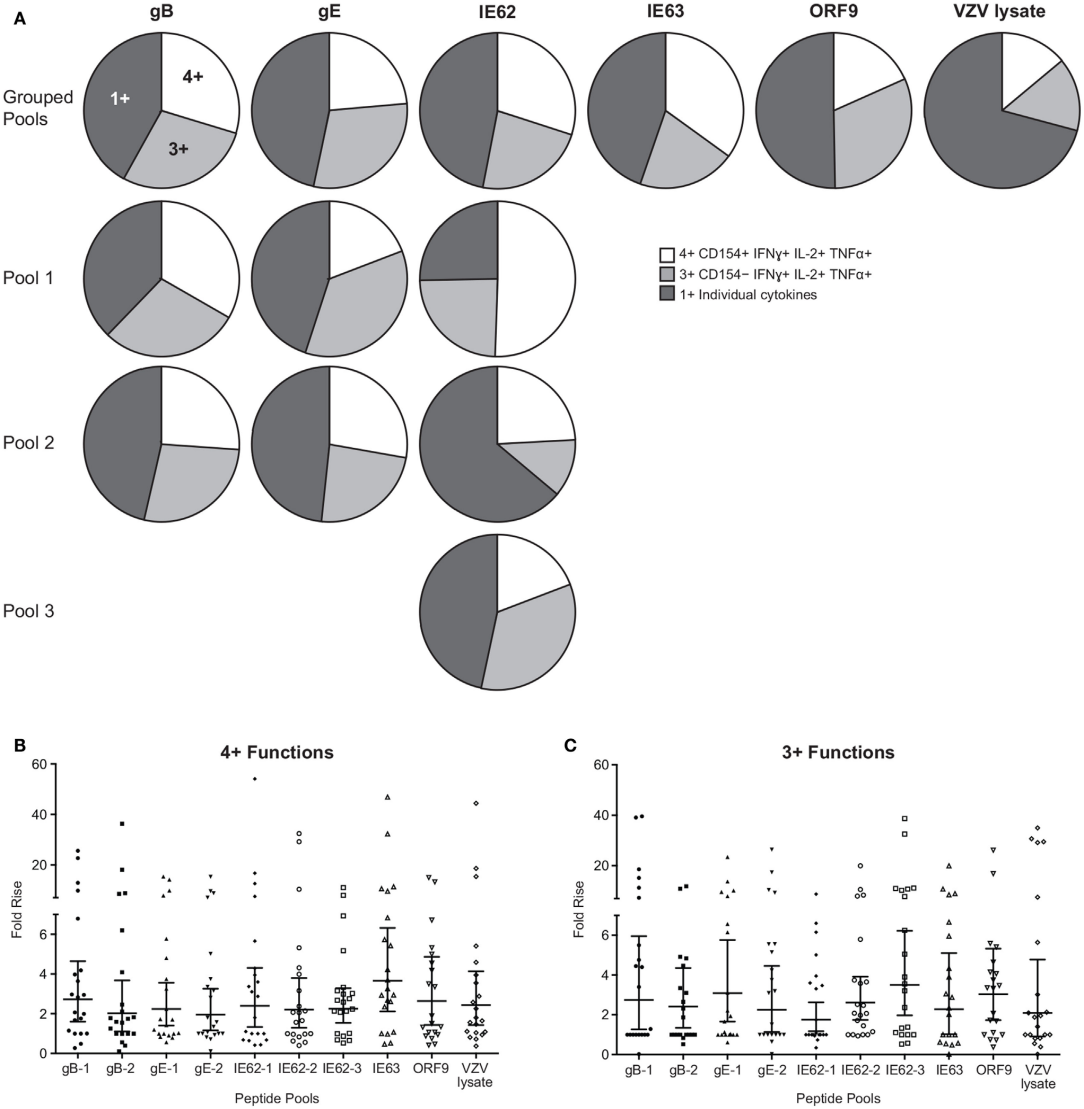
**Hierarchy of VZV antigens stimulating poly-functional CD4^+^ T cells**. **(A)** Grouped pie charts comparing the fold rise in poly-functional CD4^+^ T cells after stimulating PBMC with gB, gE, IE62, IE63, ORF9, and VZV lysate. Pie charts display 4+ functions (CD154^+^IFNγ^+^IL-2^+^TNFα^+^), 3+ functions (CD154^−^IFNγ^+^IL-2^+^TNFα^+^), or mono-functional cells. gB, gE, and IE62 were fractioned into multiple pools and poly-functional T cells were also assessed. Fold rise in the number of **(B)** 4+ (CD154^+^IFNγ^+^IL-2^+^TNFα^+^) or **(C)** 3+ (CD154^−^IFNγ^+^IL-2^+^TNFα^+^) CD4^+^ T cells for each VZV peptide pool. Each dot represents one subject, and the geometric mean ± 95% confidence interval is shown.

To examine the full complexity of the CD4^+^ T cell response, we tested whether T cells producing IFNγ, IL-2, and TNFα and expressing CD154 were poly-functional. Boolean-gating algorithm was performed, which yielded 15 unique combinations of the four measurements. Figure 3C shows IE63-specific CD4^+^ T cell responses from 21 subjects. For all antigens, a boost in the number of CD154^+^IFNγ^+^IL-2^+^TNFα^+^ (4+), and CD154^−^IFNγ^+^IL-2^+^TNFα^+^ (3+) was observed following vaccination (Figure 3C). However, minimal to no boost was observed for other functional combinations of cytokines, demonstrating that the functional heterogeneity was limited (Figure 3C). Henceforth, we only focused on 4+ (CD154^+^IFNγ^+^IL-2^+^TNFα^+^) and 3+ (CD154^−^IFNγ^+^IL-2^+^TNFα^+^) poly-functional CD4^+^ T cells and compared them to mono-functional cells (1+). By analyzing the fold rise in number of 4+ or 3+ antigen-specific CD4^+^ T cells as compared to mono-functional cells, we observed that IE63 induced the strongest poly-functional responses (Figures 4A,B). Statistical analyses ranking the five VZV antigens in fold rise of 4+ poly-functional cells demonstrated that IE63 > IE62 > gB > VZV lysate > ORF9 > gE (Table 2). Similarly, statistical analyses ranking VZV antigens in the fold induction of 3+ poly-functional demonstrated that IE62 > ORF9 > gE > gB > IE63 > VZV lysate (Table 2) and are graphically shown in (Figure 4C).

### Poly-Functional CD4^+^ T Cells Produce More of Each Cytokine on a Per-Cell Basis Than Single Producers, and an Increase in IFNγ is Observed After ZOSTAVAX Vaccination in Response to IE63 and ORF9

**Figure 5:**
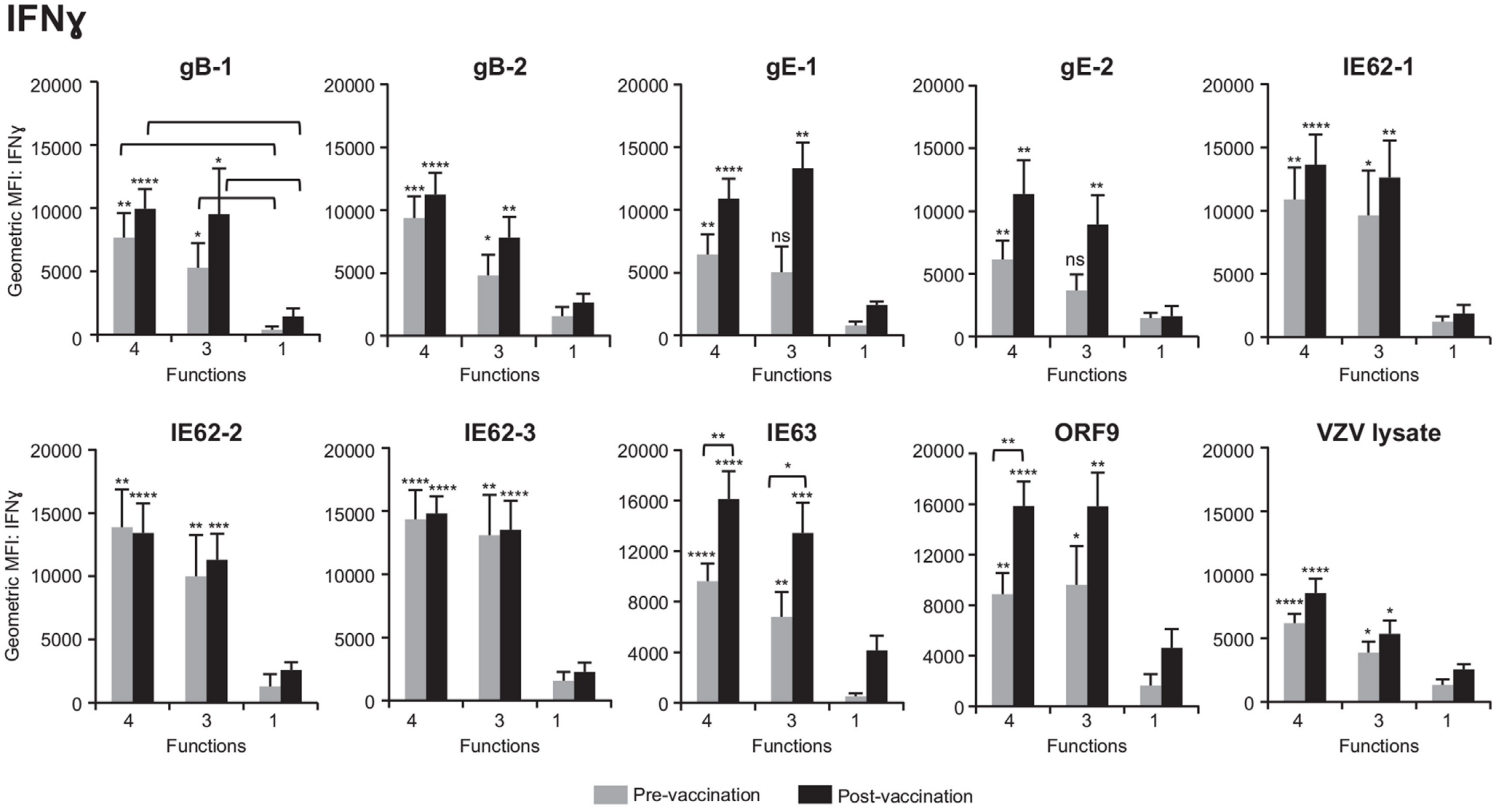
**Poly-functional CD4^+^ T cells express higher levels of IFNγ than mono-functional cells**. Geometric mean fluorescence intensity (GMFI) of IFNγ was determined in 4+ (CD154^+^IFNγ^+^IL-2^+^TNFα^+^), 3+ (CD154^−^IFNγ^+^IL-2^+^TNFα^+^), and mono-functional antigen-specific CD4^+^ T cells prior to and following ZOSTAVAX vaccination. The mean ± SEM for the 21 subjects is shown for all data sets.

We next questioned whether poly-functional CD4^+^ T cells differed in the amount of cytokine expressed on a per-cell basis as compared to mono-functional cells. To this end, geometric mean fluorescence intensity (GMFI) of IFNγ, IL-2, and TNFα was determined for each population. We found that for all antigens, prior to vaccination, 4+ and 3+ poly-functional CD4^+^ T cells produced up to 10 times more IFNγ (Figure 5), IL-2 (Figure S1A in Supplementary Material), and TNFα (Figure S1B in Supplementary Material) than mono-functional T cells. The difference in expression of IFNγ was more significant between 4+ and mono-functional cells (*P* values ≤0.001) than between 3+ and mono-functional CD4^+^ T cells (Figure 5). The same is true for IL-2 and TNFα cytokines (Figure S1 in Supplementary Material). We then assessed whether vaccination altered the cytokine expression profile of poly-functional T cells. Following vaccination, there was a statistically significant boost in the level of IFNγ production by poly-functional IE63- and ORF9-specific CD4^+^ T cells (*P* value <0.01). For other antigens, there was no statistical difference in IFNγ expression levels by poly-functional T cells, before and after vaccination. Similarly, there was no statistical difference in expression levels of IL-2 and TNFα by poly-functional T cells prior to and following vaccination. Notably, for all antigens, 4+ T cells found within the postvaccination population, produced the highest levels of IFNγ, IL-2, and TNFα with *P* values ≤0.0001 compared to mono-functional T cells.

### Poly-Functional CD4^+^ T Cells are Found Within the Central and Effector Memory Pool, and They Both Express Equivalent Levels of Cytokines

**Figure 6:**
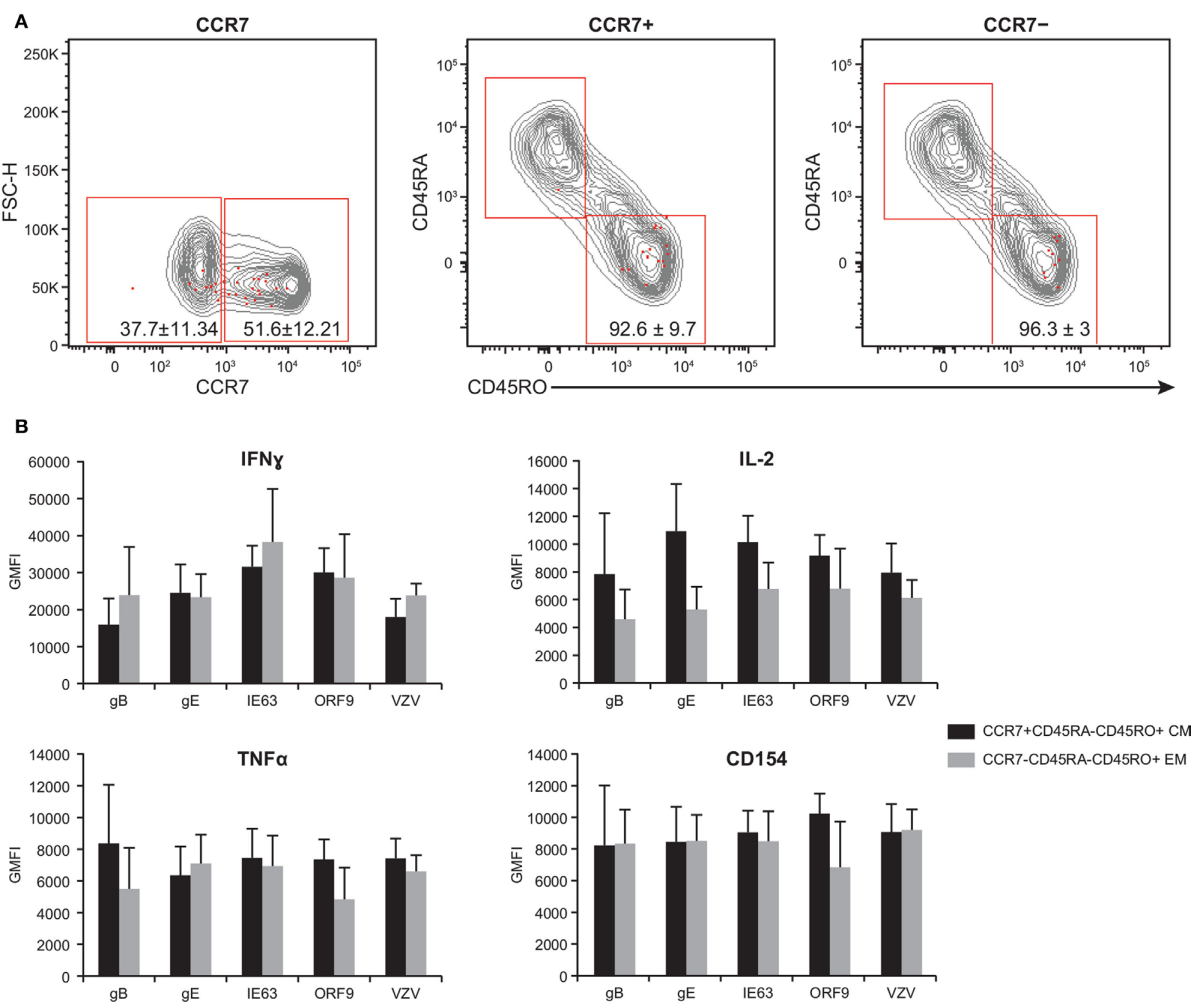
**Poly-functional CD4^+^ T cells display both T_EM_ and T_CM_ phenotypes**. PBMC isolated from five subjects following ZOSTAVAX vaccination were thawed and rested overnight before VZV antigen stimulation. PBMC were stimulated with various VZV antigens for 5.5 h at 37°C/5% CO_2_, with brefeldin A and monensin added 30 min after the initial incubation. Cells were stained with Vividye, surface stained with fluorescent primary antibodies against CCR7, CD45RA, and CD45RO. After washing off excess antibodies, PBMC were fixed, permeabilized, and stained with fluorescent primary antibodies to identify T cells producing IFNγ, IL-2, TNFα, and CD154 as described in Figure 3. Boolean analysis was performed to identify poly-functional CD4^+^ T cells with 4+ functions. **(A)** CCR7, CD45RA, and CD45RO expressions of 4+ (CD154^+^IFNγ^+^IL-2^+^TNFα^+^) antigen-specific CD4^+^ T cells (red dots) overlayed on CD3^+^ T cells (gray contours). Numbers in dot plots represent percentage mean ± SEM for the five subjects. **(B)** The GMFI of IFNγ, IL-2, TNFα, and CD154 was assessed in both T_CM_ (CCR7^+^CD45RA^−^CD45RO^+^) and T_EM_ (CCR7^−^CD45RA^−^CD45RO^+^) 4+ antigen-specific CD4^+^ T cells.

By examining the expression of CCR7, CD45RA, and CD45RO, the T cell compartment can be divided into the following memory subsets: central memory (T_CM_) (CCR7^+^CD45RA^−^CD45RO^+^), effector memory (T_EM_) (CCR7^−^CD45RA^−^CD45RO^+^), naive (CCR7^+^CD45RO^−^CD45RA^+^), and CD45RA^+^ effector memory (T_EMRA_) (CCR7^−^CD45RO^−^CD45RA^+^) (53–55). We therefore sought to investigate the phenotype of poly-functional CD4^+^ T cells responding to VZV antigens. To this end, PBMC from five subjects were stimulated with various peptide pools than stained for the expression of the CCR7, CD45RA, and CD45RO, concurrently with IFNγ, IL-2, TNFα, and CD154. These five subjects were chosen due to the availability of enough PBMC to carry out the entire set of analyses. Furthermore, these subjects showed similar phenotype and robust T cell responses to various antigens, and hence, they served as good representatives of the 21 subjects. Figure 6A shows the expression of the memory markers by antigen-specific CD4^+^ T cells (red dots) over total CD3^+^ T cells (gray contours). We found a trend toward higher frequency of 4+ poly-functional cells T_CM_ (51.6% ± 12.21, mean ± SEM) expressing CCR7^+^, as compared to 37.7% ± 11.34 CCR7^−^ T_EM_ cells. Of the CCR7^+^ population, 92.6% ± 9.7 was CD45RA^−^CD45RO^+^, while 96.3% ± 2.9 of the CCR7^−^ subset was CD45RA^−^CD45RO^+^ (Figure 6A). Notably, neither naive nor T_EMRA_ was found within the 4+ poly-functional CD4^+^ T cell population (Figure 6A). Functional assessment of IFNγ, IL-2, TNFα, and CD154 expression by T_CM_ versus T_EM_ 4+ poly-functional CD4^+^ T cells revealed that both memory populations produced equivalent levels of IFNγ, IL-2, and TNFα cytokines (Figure 6B).

### Fold Rise in Central and Effector Memory Poly-Functional ORF9-Specific CD8^+^ T Cells Following ZOSTAVAX Vaccination

**Figure 7:**
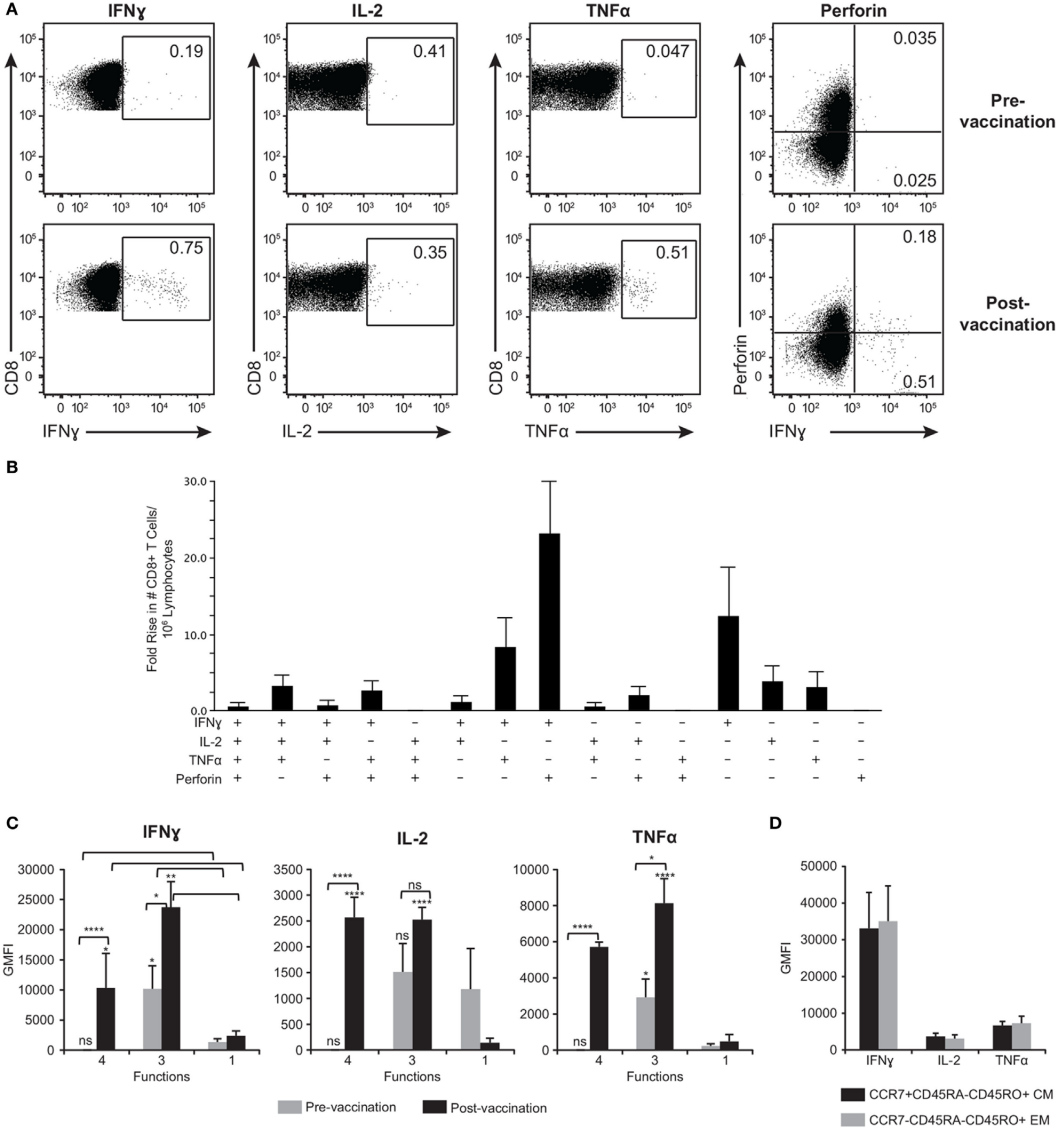
**ORF9-specific CD8^+^ T cells are poly-functional, produce more cytokines than mono-functional cells, and display both T_EM_ and T_CM_ phenotypes**. **(A)** Representative gating strategy of PBMC prior to and following ZOSTAVAX vaccination that were stimulated with ORF9 peptide pool. Numbers in dot plots denotes percentages of CD8^+^ T cells producing IFNγ, IL-2, TNFα, and perforin. **(B)** Fold rise in the number of poly-functional CD8^+^ T cells demonstrating the heterogeneity of the T cell population. **(C)** The expression levels (GMFI) of IFNγ, IL-2, and TNFα by 4+ (IFNγ^+^IL-2^+^TNFα^+^perforin^+^), 3+ (IFNγ^+^IL-2^+^TNFα^+^), and mono-functional cells prior to and following ZOSTAVAX vaccination. **(D)** GMFI of IFNγ, IL-2, and TNFα was assessed in both ORF9-specific CD8^+^ T_CM_ (CCR7^+^CD45RA^−^CD45RO^+^) and T_EM_ (CCR7^−^CD45RA^−^CD45RO^+^).

While we were able to detect CD4^+^ T cell responses after activation with gB, gE, IE62, IE63, and ORF9, detection of CD8^+^ T cell responses was limited to ORF9 activation, with 13 of 21 (62%) subjects responding. Figure 7A is representative data showing ORF9 stimulation of PBMC isolated before and after ZOSTAVAX vaccination in one subject. In addition to quantification of IFNγ, IL-2, and TNFα production by CD8^+^ T cells, we also assessed *de novo* expression of perforin by activated IFNγ^+^ T lymphocytes denoting cytotoxic T lymphocytes (56) (Figure 7A). Prior to vaccination, a low frequency of memory CD8^+^ T cells producing IFNγ, IL-2, and TNFα was detected (Figure 7A). Postvaccination, the median frequency of CD8^+^ T cells producing IFNγ^+^ increased by 2.8-fold, and TNFα^+^ increased by 4.9-fold (Figure 7A). The frequency of IL-2^+^ CD8^+^ T cells did not change after vaccination (Figure 7A). Notably, IFNγ^+^perforin^+^ CD8^+^ T cells were only observed after vaccination (Figure 7A). We observed that the functional heterogeneity of the ORF9-specific CD8 T cells was quite diverse. The CD8^+^ T cells that had the highest fold increase (~20-fold) after vaccination were IFNγ^+^perforin^+^ cells followed by IFNγ^+^ cells (Figure 7B). Albeit rare, after ZOSTAVAX vaccination we were able to identify CD8^+^ T cells displaying all 4+ functions (IFNγ^+^IL-2^+^TNFα^+^perforin^+^) (Figure 7B). We also observed a threefold increase in the frequency of 3+ T cells expressing IFNγ, IL-2, and TNFα after vaccination (Figure 7B). Examining cytokine expression levels, both 4+ and 3+ CD8^+^ T cells prior to vaccination expressed low levels of IFNγ, IL-2, and TNFα that were comparable to mono-functional cells (Figure 7C). After vaccination, both 4+ and 3+ produced ~10-fold higher levels of IFNγ, IL-2, and TNFα than 1+ ORF9-specific CD8^+^ T cells (Figure 7C) and demonstrated a statistically significant boost (*P* ≥ 0.0001) in the expression of IFNγ, IL-2, and TNFα cytokines compared to prevaccination samples (Figure 7C). As with the CD4 T cell responses, we found a slightly higher proportion of 3+ poly-functional CD8^+^ T cells that were T_CM_ (48.8% ± 11.2) versus 43.4% ± 12.8 T_EM_. These data demonstrate that ORF9-specific CD8^+^ T cell populations were dominated by both T_CM_ and T_EM_. We observed no difference in the expression levels of IFNγ, IL-2, and TNFα (Figure 7D).

## Discussion

Previous studies investigating T cell responses to ZOSTAVAX demonstrated an increase in IFNγ ELISPOT responses (9, 13, 26) and effector memory T cells (9), thereby demonstrating that IFNγ-dependent T cell-mediated immune responses may contribute to ZOSTAVAX efficacy. A recent study by Laing et al. (28) surveyed the whole VZV genome for CD4^+^ reactivity by assessing the proliferation of VZV-specific T cell lines from 12 ZOSTAVAX recipients. They showed that responses to 42 VZV ORF were boosted upon vaccination (28). Indeed, we confirm their findings by reporting that cell-mediated responses against gB, gE, IE61, IE63, ORF4, ORF9, ORF10, ORF19, and ORF29 were observed. Notably, the 10 VZV antigens investigated in our study were within the top 50% of the ORF reported by Laing et al. However, we did not want to introduce the bias of ORF expression and antigen presentation *in vitro* by the generation of T cell lines and hence used peptide stimulation *ex vivo* to get unbiased sampling of memory T cell responses. Between the two studies, the rank order for the magnitude of the responses to individual antigens differs as our approach of using peptides to assay T-cell responses avoids the bias of antigen expression, processing for presentation, and selective expansion of T cells. Irrespective of the methods used, the independent corroboration of similar VZV antigens gives credence to the observation.

Compelling evidence demonstrating that poly-functional T cells producing IFNγ, IL-2, and TNFα correlate with protection was shown using the mouse model of *Leishmania major* and *Mycobacterium tuberculosis* infection (57). Indeed, multifunctional T cells have also been shown to be beneficial in human infections, such as HIV (58) and cytomegalovirus (59), and following vaccination with vaccinia (60, 61), hepatitis B, and tetanus toxoid (30). Among a diverse heterogeneous population of CD4^+^ T cells responding to various VZV antigens, we observed that the highest frequency of cells was CD154^+^IFNγ^+^IL-2^+^TNFα^+^ (4+ functions) and CD154^−^IFNγ^+^IL-2^+^TNFα^+^ (3+ functions) T cells. A previous study investigating CD4^+^ T cell responses after vaccination with HZ/gE subunit vaccine observed that the dominant population of T cells that increased following vaccination was CD154^+^IL-2^+^, and to a lesser extent IFNγ or TNFα expressing cells (62). By contrast, our study demonstrated a minimal increase in the frequency of gE-specific CD154^+^IL-2^+^ CD4 T cells and a higher frequency of 4+ CD4^+^ poly-functional T cells after vaccination. This discordance could be attributed to the use of different types of vaccines (subunit versus live attenuated). More importantly, the frequency of 4+ poly-functional CD4^+^ T cells was the highest among all phenotypes for the VZV antigens tested, demonstrating that this population is the dominant responder. In agreement with a previous study, we also observed poly-functional CD4^+^ T cells with three functions (IFNγ^+^IL-2^+^TNFα^+^) that were reactive to VZV lysate (11). However, we found that only 30% of CD4^+^ T cells responding to VZV lysate were poly-functional. While the larger proteins provided numerous epitopes for T cell activation, we found that length of the protein was not a factor as demonstrated by robust CD4^+^ reactivity to ORF9 (302 amino acids) and IE63 (278 amino acids).

Prior investigations have demonstrated a skewing of anti-VZV T cell responses toward CD4^+^ T cells (11, 14, 15, 18–20, 22, 23, 28, 41, 43–45, 63), with few VZV-specific CD8^+^ T cells reported (9, 14, 17, 20, 44, 63). By following standard intracellular cytokine staining (ICS) protocols, the frequency of VZV-specific CD8^+^ T cells was shown to be below the limit of detection (17, 21, 62). Therefore, to detect VZV-specific CD8^+^ T cells, rigorous activation protocols have been utilized such as overnight stimulation of PBMC with live VZV (9) and the activation of PBMC for several days with either VZV lysate or peptides prior (17, 20, 44, 63). The extended incubation of PBMC with VZV facilitates the processing and presentation of antigen to CD8^+^ T cells and generation of *de novo* antigen-specific T cells. However, previous studies that stimulated PBMC with peptides which do not require processing were unable to detect antigen-specific CD8^+^ T cells (17). In agreement with the previous studies, we found that the frequency of gB, gE, IE62, and IE63-specific CD8^+^ T cells was below the limit of detection via ICS. Surprisingly, we were able to detect *ex vivo* poly-functional CD8^+^ T cells that were reactive to ORF9 in 62% of the subjects. Whether these cells play a role in cytolysis of virus-infected cells remains to be elucidated; however, their expression of IFNγ, IL-2, TNFα, and perforin suggests that they are indeed armed for this function. Moreover, the induction of poly-functional CD8^+^ T cells suggests that ZOSTAVAX replicates *in vivo*.

The benefit of poly-functional T cells in protection against pathogens has been attributed by their: (a) ability to express higher levels of IFNγ on a per-cell basis than other populations (49, 57, 60, 64); (b) enhanced cytotoxicity due to secretion of both IFNγ and TNFα (65, 66); and (c) high IL-2-mediated proliferative capacity (67). Indeed, our investigations demonstrate that on a per-cell basis VZV-specific poly-functional CD4^+^ and CD8^+^ T cells produce 10-fold more IFNγ, IL-2, and TNFα than mono-functional T cells. A marked increase in IFNγ expression by poly-functional IE63- and ORF9-specific CD4^+^ T cells was observed after vaccination. The difference in expression of IFNγ, IL-2, and TNFα cytokines was more significant between 4+ and mono-functional cells than between 3+ and mono-functional CD4^+^ T cells. After ZOSTAVAX vaccination, a marked increase in IFNγ, IL-2, and TNFα cytokine expression levels was also observed by CD8^+^ poly-functional T cells.

Given that no other reports had detected *ex vivo* antigen-specific CD8^+^ T cells without extended T cell culture, our identification of ORF9-specific CD8^+^ T cells was quite surprising. Interestingly, crossreactive human CD8^+^ T cells that recognize conserved epitopes among related herpes viruses have been reported (68). The authors found a CD8^+^ T cell epitope from VZV ribonuclease reductase subunit 2 protein (ORF18) that was homologous to HSV and Epstein–Barr virus (68). However, ZOSTAVAX vaccination was not reported to stimulate/enhance ORF18 epitope-specific CD8^+^ T cell responses in majority of the subjects (68). Following the same rationale, we questioned whether ORF9-specific CD8^+^ T cell responses observed in our study were derived from crossreactivity with HSV. ORF9 protein is an ortholog of HSV VP22 protein that shares 25% identity and 34% similarity (69) and is the most abundant VZV transcript during primary infection (51). VP22 is encoded by UL49 gene, and as such, ORF9 gene is 43% identical and 56% similar to UL49 (69). Interestingly, poly-functional UL49-specific CD8^+^ T cells in HSV-infected individuals have been shown to produce IFNγ, IL-2, and TNFα (49). Therefore, to assess this crossreactive possibility, PBMC from HSV-positive donors were stimulated with UL49 and ORF9 peptide pools, and ICS performed to detect IFNγ, IL-2, and TNFα production by CD4^+^ and CD8^+^ T cells. While UL49-specific CD4^+^ and CD8^+^ T cells producing IFNγ, IL-2, and TNFα could be detected, we could not detect ORF9-specific T cells (data not shown). In a converse experiment, ZOSTAVAX vaccines with ORF9-specific CD8^+^ and CD4^+^ T cell responses were stimulated with UL49 peptide pool. Notably, no UL49-specific CD8^+^ responses were detected, whereas two of the five subjects showed UL49-specific CD4^+^ T cell responses. The latter CD4^+^ T cell results are confounded by the fact that we did not type our ZOSTAVAX vaccines for HSV exposure. These data led us to infer that the ORF9-specific CD8^+^ T cells responses were not due to crossreactivity to HSV.

In conclusion, our evaluation of VZV-specific T cell-mediated immune responses demonstrated that a wide repertoire of both T_CM_ and T_EM_ poly-functional CD4^+^ T cells expressing IFNγ, IL-2, TNFα, and CD154 were boosted following ZOSTAVAX vaccination. Poly-functional IE63-specific CD4^+^ T cells were the dominant responders as evidenced by a statistically significant boost in cell frequency and the level of IFNγ produced on a per-cell basis. A novel ORF9-specific poly-functional CD8^+^ T cells was also boosted upon vaccination, expressed higher levels of cytokines following vaccination, and was found to belong to both T_CM_ and T_EM_ population.

## Author Contributions

Conceived and designed of the experiments: JS, KC, SD, DK, DC, and KV. Performed the experiments: JS, KC, and SD. Analyzed or interpreted the data: JS, KC, SD, JA, and KV. Wrote the manuscript: JS and KV.

## Conflict of Interest Statement

All authors are employees of Merck & Co., Inc. and may hold stock in the company as a part of their annual compensation.
