# Supplementary figures and images for: Effector and Central Memory Poly-Functional CD4+ and CD8+ T Cells are Boosted upon ZOSTAVAX® Vaccination
Source: Front Immunol. 2015 Oct 29;6:553. doi: 10.3389/fimmu.2015.00553 (PMC4629102; doi:10.3389/fimmu.2015.00553)

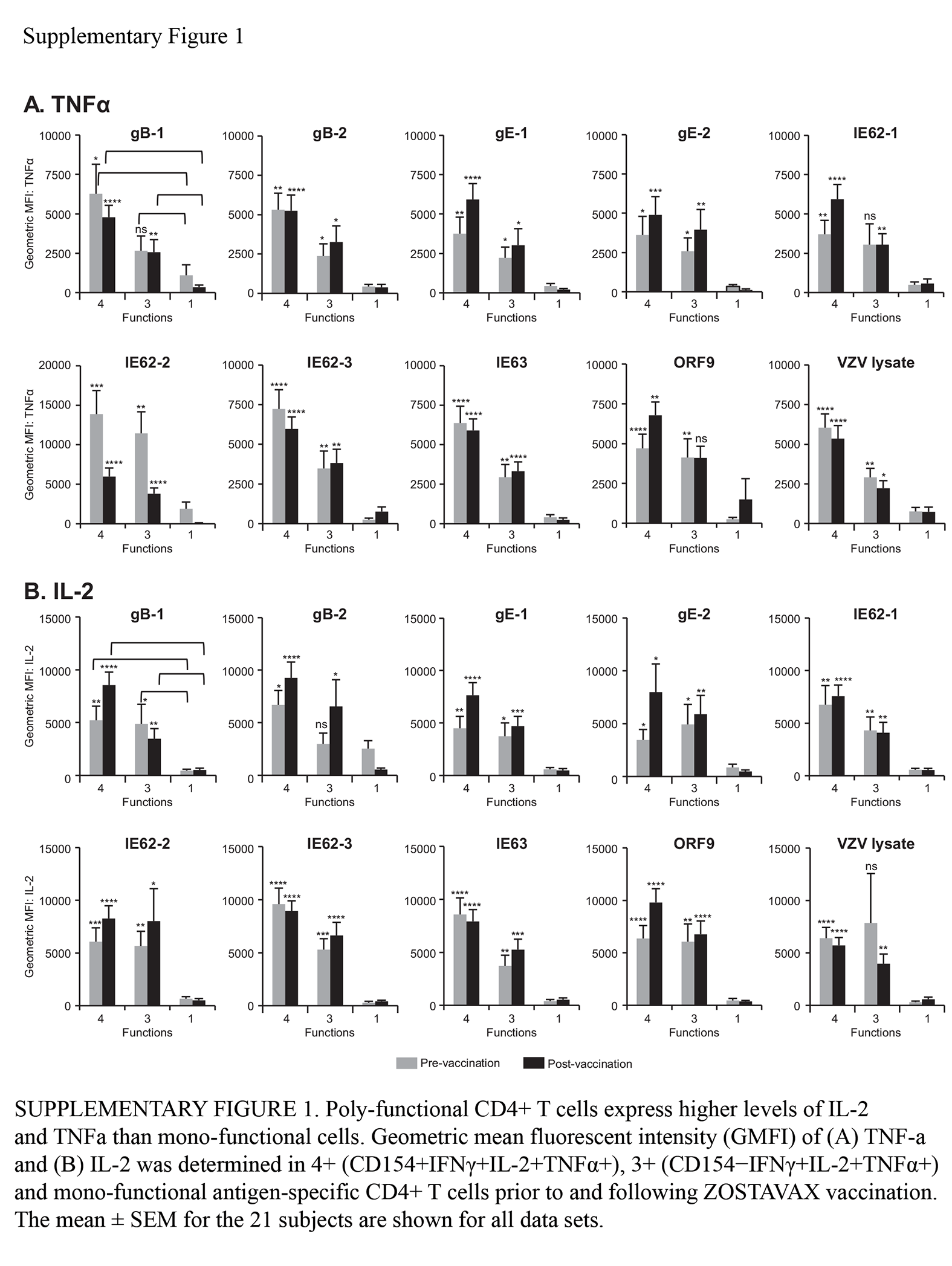

Supplement: Supplementary file 1 [file Image_1.TIF]
